# Big Data Technology Applications and the Right to Health in China during the COVID-19 Pandemic

**DOI:** 10.3390/ijerph18147325

**Published:** 2021-07-08

**Authors:** Taixia Shen, Chao Wang

**Affiliations:** 1 Law School & Intellectual Property School, Jinan University, Guangzhou 510632, China; sunbird726@hotmail.com; 2 Faculty of Law, University of Macau, Taipa, Macao 999078, China

**Keywords:** big data technology, COVID-19, epidemic prevention and control, human rights, right to health, right to privacy, China

## Abstract

Individuals have the right to health according to the Constitution and other laws in China. Significant barriers have prevented the full realisation of the right to health in the COVID-19 era. Big data technology, which is a vital tool for COVID-19 containment, has been a central topic of discussion, as it has been used to protect the right to health through public health surveillance, contact tracing, real-time epidemic outbreak monitoring, trend forecasting, online consultations, and the allocation of medical and health resources in China. Big data technology has enabled precise and efficient epidemic prevention and control and has improved the efficiency and accuracy of the diagnosis and treatment of this new form of coronavirus pneumonia due to Chinese institutional factors. Although big data technology has successfully supported the containment of the virus and protected the right to health in the COVID-19 era, it also risks infringing on individual privacy rights. Chinese policymakers should understand the positive and negative impacts of big data technology and should prioritise the Personal Information Protection Law and other laws that are meant to protect and strengthen the right to privacy.

## 1. Introduction

The new coronavirus pneumonia (COVID-19) has struck with unprecedented scale and ferocity. Statistics show that, as of 31 May 2021, the total number of confirmed COVID-19 cases had reached 170,051,718 worldwide, with 3,540,437 deaths [1]. It is difficult to cope with such a major public health crisis with limited medical and health resources in every state. In response to its outbreak, China adopted extensive, stringent, and thorough containment measures to prevent and control the COVID-19 pandemic and protect citizens’ right to health.

Data analysis, algorithms, and mathematical modelling are increasingly shaping health policy responses. From surveillance mapping to mobile phone apps, new technologies are being woven into diagnostic and therapeutic services on many levels. These technologies and tools offer advances in health care and are being used in pandemic prevention and control worldwide [2,3].

Developments in science and technology have always gone hand in hand with the history of humankind, but the awareness of their potential impacts on human beings is closely linked to the rise of human rights as deontic properties in the legal field [4,5]. These developments raise crucial questions. Existing literature points out the human rights concerns of big data applications [6,7]. How should big data technology be used to realise and protect the right to health? Is the application of big data technology effective in protecting the right to health? What are the risks and consequences of this kind of technology? This study investigated China’s application of big data technology to protect its citizens’ right to health during the COVID-19 prevention and control period and explored the positive and negative impacts of this technology.

The remainder of this paper is organised as follows. Section 2 introduces the legal institution underpinning the right to health in China. Section 3 highlights the main obstacles to the realisation of the right to health during the COVID-19 pandemic. Section 4 explores how the right to health can be protected using big data technology during the COVID-19 pandemic. Section 5 examines the positive and negative impacts of big data technology applications. Section 6 discusses the main institutional causes of the impacts of big data technology and Section 7 draws conclusions based on the findings of this study.

## 2. Legal Institutional Framework of Right to Health in China

The right to health was first articulated as a human right in the 1946 Constitution of the World Health Organisation and was further recognised in Article 25 of the Universal Declaration of Human Rights, Articles 9–12 of the International Covenant on Economic, Social and Cultural Rights (ICESCR), and other international and regional treaties. Article 12 of the ICESCR recognises ‘the right of everyone to the enjoyment of the highest attainable standard of physical and mental health’. General Comment No. 14 of the United Nations Committee on Economic, Social and Cultural Rights summarises the principles and resources required to fulfil the right to health. The right to health necessarily connotes certain freedoms and entitlements. Such freedoms include the right to be free from non-consensual medical treatment, torture, and other cruel, inhumane, or degrading treatment or punishment. The entitlements include the right to a system that provides (1) equality of health protection for everyone; (2) the prevention, treatment, and control of diseases; (3) access to essential medicines; (4) maternal, child, and reproductive health; (5) equal and timely access to basic health services; (6) health-related education and information; and (7) opportunities for citizens to participate in health-related policy and decision making at the national and community levels [8,9]. Other international human rights treaties have recognised or referred to the right to health and its related aspects as the right to medical care. However, the specifics of the right to health vary across regional human rights instruments and national constitutions. Most national constitutions set out duties to respect, protect, and fulfil the right to health.

The Constitution of the People’s Republic of China (the PRC Constitution) does not specify health as a fundamental right, but Articles 21 and 45 of the PRC Constitution stipulate that the state is obligated to protect its citizens’ health. Article 26 stipulates that the state is obligated to prevent and control pollution and other public hazards. Article 36 stipulates that citizens’ health is inviolable. The Promotion of Basic Medical and Health Care Law of the People’s Republic of China (‘the PRC Health Promotion Law’) clarifies the legal status of the right to health and the state’s obligation to respect and protect the right to health, with Article 5 stipulating that individuals should have access to basic medical and health services.

According to the PRC Health Promotion Law, individuals have the right to basic medical and health services from the state. Article 15 of this law defines basic medical and health services as including disease prevention, diagnosis, treatment, nursing, rehabilitation, and other services provided through drugs, technology, and suitable equipment required for the preservation of individuals’ health within the state’s economic development and capacities and stipulates that they should be equitably available to all. Article 4 also stipulates the right to health education.

The legal theory and international covenants all embody several elements: medical and health services, goods, and facilities must be provided to all without discrimination, and all medical and health services, goods, and facilities must be available, accessible, medically and culturally acceptable, scientifically and medically appropriate, and of good quality. To achieve this, functioning public health and healthcare facilities, goods, and services must be available in sufficient quantity within a state, and they must be physically and financially accessible to everyone without discrimination [8,9]. Articles 15 and 39 of the PRC Health Promotion Law also stipulate that basic medical and health services should be equitably available and accessible.

**Figure 1:**
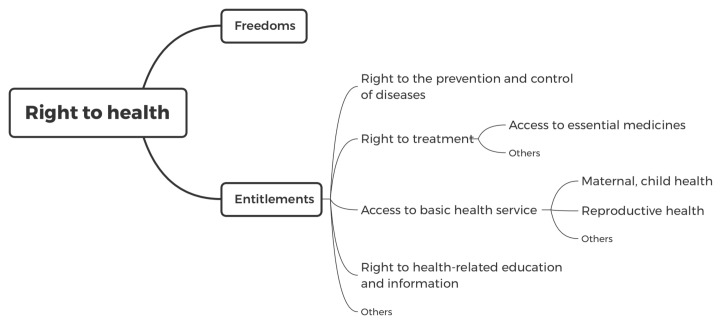
Conceptual system of the right to health in China.

According to the legal institution that underpins the right to health, many Chinese scholars believe that the right to health does not guarantee the right of individuals to be healthy, but it encompasses several freedoms and entitlements to enable individuals to attain the highest standard of health possible [10]. The conceptual system of the right to health is illustrated in Figure 1.

## 3. Obstacles to Realising the Right to Health during China’s Epidemic Prevention and Control

The PRC Health Promotion Law includes institutional arrangements to protect and fulfil Chinese citizens’ right to health. The obligations of the state and society include the rational planning and allocation of medical and health resources; increased financial inputs into the medical and healthcare sectors, including the establishment and improvement of a medical service system composed of grassroots medical institutions, hospitals, and professional public health institutions; and the establishment of a multi-layered social security system to realise citizens’ right to health. During the epidemic prevention and control period, both the state and society are obligated to guarantee and provide healthcare services to citizens. However, in the COVID-19 era, significant barriers prevent the full realisation of the right to health.

### 3.1. The Right to the Prevention and Control of Diseases

Preventing and controlling COVID-19 and other epidemic diseases requires basic epidemic prevention supplies, such as masks and disinfectants, which play a key role in ensuring the right to health and preventing the spread of the virus. Individuals also have an equal right to be vaccinated when a vaccine becomes available. The state is therefore obligated to adequately supply basic public health and epidemic prevention products to meet individuals’ needs.

However, to achieve this right to the prevention and control of epidemics, a healthy public health environment that is free of virus contamination is essential. The government and its relevant entities can only guarantee this through the on-site control, disinfection, and isolation of public places. The government is therefore obligated to set up isolation places, conduct epidemic detection at major traffic stations, and sterilise public places. Additionally, the state is obligated to report, monitor, investigate, and conduct epidemiological investigations on COVID-19 infections to contain the source of the infection.

For various reasons, some aspects of the right to health were not guaranteed in the early stage of epidemic prevention and control. First, obtaining basic epidemic prevention supplies was impossible in some cases because of severe shortages. In the early stage of epidemic prevention and control, the high demand for personal protective equipment, such as gloves, face masks, goggles, face shields, and gowns, led to severe shortages of emergency medical materials in Hubei province and other places [11]. The frontline medical staff were limited by the government due to insufficient protective supplies, and to save time and protective supplies, healthcare providers did not eat or drink to avoid going to the restroom during working hours [12]. Amid the emergency and medical material shortage caused by the COVID-19 outbreak, China built the 1000-bed Huoshenshan Hospital in Wuhan and completed the construction of the Leishenshan Hospital, a second 1600-bed facility, in just a few days [13].

Second, access to a healthy public environment is constrained by a country’s public health security system and its ability to respond to major public health emergencies. Since the end of 2019, COVID-19 has swept the world, and many countries’ public health systems were shown to be inadequate at preventing the spread of the disease. In the early stage of epidemic prevention and control, China’s response could not keep pace with the rapid spread of the virus, largely because outbreak information was often not reported promptly. Low detection capacity and insufficient isolation locations were major deficiencies, indicating the need to improve China’s capacity to prevent, detect, and respond quickly to public health emergencies. Before the COVID-19 outbreak, the Johns Hopkins Center for Health and Safety, in collaboration with the Economist Intelligence Unit, published the Global Health and Safety Reporting Index 2019, which documented significant problems with public health security in most countries. The average overall Global Health and Safety Index score of the 195 countries assessed was a mere 40.2 out of a possible 100, and China’s overall score was 48.2 [14]. As indicated, China has deficiencies in its national capacity to prevent, detect, and respond to health emergencies, as do the country’s sub-regional public health systems.

### 3.2. The Right to Treatment

Although the diagnosis and treatment options for COVID-19 patients differ from those of other types of patients, both cohorts have an equal right to treatment. Patients infected with SARS-CoV-2 have the right to obtain a timely and effective diagnosis, treatment, and rehabilitation. Patients with COVID-19 are triaged using four classifications (i.e., light, normal, heavy, and critical), and different patients have different treatments and rehabilitation programmes [15]. Severe and critically ill patients are at risk of dying when they cannot be treated quickly and effectively. Likewise, patients who are not infected with SARS-CoV-2 have the right to obtain a timely and effective diagnosis, treatment, and rehabilitation during the epidemic prevention period. A patient with an acute disease, such as a cerebral haemorrhage, may face a life-threatening medical situation if they do not receive timely and effective treatment.

Basic medical treatment is typically divided into general and emergency services. Patients can only utilise their right to access treatment within these two categories. To effectively guarantee patients’ right to treatment, the state is obligated to provide adequate medical facilities for citizens, to provide medical services with adequate medical personnel, and to provide basic medical supplies. Chinese medical insurance has improved substantially since the establishment of basic medical insurance for urban employees in 1998. In addition, to guarantee the implementation of this right, China has established a broad medical insurance system and a medical assistance system to provide humanitarian assistance to individuals who are unable to obtain medical insurance since 2013 [16,17].

During the epidemic prevention and control period, the realisation of citizens’ right to treatment faced many difficulties. First, patients’ right to COVID-19 treatment was restricted by an insufficient supply of basic medical resources. There were insufficient infectious disease hospitals and isolation wards, inadequate professional care, and insufficient medication for treating patients with COVID-19. According to the 2019 China Health Statistics Yearbook, the number of infectious disease hospitals in China in 2018 was 167, but there were 338 prefecture-level cities, more than half of which did not even have one such hospital [18]. At the beginning of the COVID-19 outbreak, Huanggang city in Hubei province, the area with the second-largest number of COVID-19 cases in China, was unable to set up a qualifying infectious disease hospital by 21 January 2020 [19]. In Wuhan, the virus spread quickly, forcing the city to lock down for 76 days. By mid-March, 42,600 healthcare professionals from other regions in China were “relocated” to Wuhan to overcome the shortage of local professional healthcare [20].

Second, during the SARS-CoV-2 outbreak, ordinary patients’ right to treatment was affected by the reallocation of basic medical resources. Patients with chronic diseases were not treated promptly, as hospitals around the country closed general departments to concentrate their limited resources on epidemic prevention and control. Many ordinary patients were unable to go to a hospital to receive treatment [21,22]. Between the end of January and mid-March, healthcare services (except emergency care) were largely paused [20,22].

All patients should have ready access to essential medicines for timely and effective treatment. As the right to health is constrained by a country’s economic development and medical and healthcare systems, it is pragmatically restricted to essential medicines. Both COVID-19 patients and other patients need to obtain essential medicines. There is no special medication to treat the symptoms of COVID-19 because medical knowledge and research on COVID-19 are still very limited. However, many different treatment options have been proposed, and some older drugs seem to be associated with positive outcomes [12,23]. However, the long-term strategy for combatting COVID-19, which has spread to nearly every country in the world, is to launch vaccination programmes.

### 3.3. The Access to Basic Health Services

China’s basic public health services are broad in scope, encompassing the original basic public health services, such as the establishment of resident health files, health education, vaccination, child health management, maternal health management, infectious disease and public health emergency reporting and handling, and health supervision and co-management [24]. All of these are encapsulated in law under Article 49 (3) of the Emergency Response Law of the People’s Republic of China, Article 34 of the Emergency Regulations on Public Health Emergencies of the People’s Republic of China, the National Code of Basic Public Health Services of People’s Republic of China (Third Edition), and others. The right to equal and timely basic health services is necessary for individuals to maintain their health. The development of the right to basic health services is consistent with the country’s economic development level and medical and health conditions. Given the economic and practical constraints of providing various health services, ‘basic’ in this context is generally taken to mean ‘necessary to maintain human health’—namely, to protect citizens’ lives. To fulfil the right to basic health services, the state is obligated to provide adequate health facilities for citizens and sufficient healthcare workers, services, and supplies. Such basic services are usually obtained at public hospitals in China. However, many healthcare centres were temporarily closed so that medical resources could be concentrated on the prevention and treatment of COVID-19. Official statistics show that nationwide healthcare service provisions from January to April of 2020 decreased by about 26.1% compared with the same period in 2019 due to the impact of COVID-19 [25]. There were reports of patients having to delay necessary health care, such as surgery and dialysis, due to the disruption of normal healthcare services [26].

### 3.4. Right to Health-Related Education and Information

Given the prevalence of COVID-19 and the fact that many people still do not understand the nature and characteristics of the disease, the government is obligated to provide the public with basic information and knowledge about the virus and other related issues, such as the means of preventing the transmission of the virus. Such health-based education plays an important role in limiting the spread of the disease. Article 10 of the PRC Prevention and Treatment of Infectious Diseases Law stipulates that the state must institute health education for the prevention of infectious diseases. The government, media, schools, disease prevention and control entities, and medical institutions must all be involved in such campaigns.

However, citizens’ information and knowledge regarding preventing epidemics are restricted by limitations on information and cognitive biases. COVID-19 differs from SARS and other respiratory diseases; in the early stages of the outbreak, the public lacked sufficient information about COVID-19. People did not know that the virus was extremely infectious, they were not aware of how to prevent and control infection, and many were panic-stricken. Some studies showed that even professional medical personnel had a worrisome lack of knowledge about COVID-19 in the early stages of the outbreak [12,27].

When providing basic public medical and health services, the government should meet the requirements of equality, availability, accessibility, acceptability, and appropriateness. In response to major public health emergencies, the government should institute measures to increase the supply of public health and epidemic prevention materials to increase the public’s health and epidemic prevention knowledge. The distribution of public health materials and services should prioritise the most urgent needs, with special attention being given to the most vulnerable groups [28].

## 4. How to Protect the Right to Health with Big Data Technology in China during the COVID-19 Pandemic

Big data has long been used for business applications, but the technology is rapidly being extended to other fields, including the manufacture of health care machines, social media, and satellite imaging [29]. The core function of big data technology is to predict future trends from existing patterns, which necessitates extensive information collection and technology to process and analyse the massive stores of data. China first established an automatic early warning and information surveillance system for national infectious diseases in April 2008, and a data monitoring system for infectious diseases was instituted in December 2009 [30]. Thus far, big data has mostly been applied in the fields of telecommunications, aerospace engineering, medical treatment, and even government policy. The application of big data in the field of public health is now attracting more attention because of COVID-19. In addition to making diagnoses, big data is becoming prominent in sensitive and specific infectious disease monitoring and early warning systems based on infectious disease monitoring data.

In China, the use of big data technology for the prevention and control of COVID-19 includes data collection, analysis, and application. First, the central government, local governments, public institutions, residents’ committees, villagers’ committees, and companies collect location, travel, health status, medical, government, consumption, media, and other data by accessing telecommunication, internet, electronic medical records, hospital and government information systems, epidemic prevention systems, smart devices, questionnaire results, map company information, and appointment registration dates. This information is then studied using descriptive, diagnostic, predictive, and prescriptive analytics for personal tracking, and the results can then be used for epidemic surveillance, early warning, tracking of virus sources, drug screening, medical treatment, resource allocation, and production recovery [31]. This section focuses on how to protect the right to health through the application of big data technology.

### 4.1. Protecting the Right to Prevention and Control of Epidemic Diseases with Tracking and Epidemic Surveillance Technology

Compared with SARS, China has used big data extensively to increase the effectiveness of protecting the right to prevention and control of epidemic diseases.

Epidemiology is one of the most important branches of data science. In terms of epidemic prevention and control, data science not only concerns collecting statistics on daily outbreaks but is also an important means of understanding the patterns of infection, epidemic rules, and mitigation strategies. Using a Social Media Search Index to predict outbreaks of COVID-19 in affected areas could be effective at protecting the right to prevention and control of epidemic disease, as it has demonstrated a high correlation with new suspected and confirmed COVID-19 infection cases [31].

First, big data technology is used for epidemic monitoring and analysis, which provides an empirical basis for the health sector to make decisions. Through search engine data, data mining, and data analysis, relevant information can be extracted to determine and predict the occurrence and development trends of diseases. In March 2020, the Chinese government implemented a national monitoring system using mobile phone positioning data to generate a quick medical response code that indicates an individual’s health status. To obtain a code, citizens must visit the sign-up page and provide their personal information, including their name, national identity number or passport number, and phone number. Applicants must also report their travel history and any possible contact with a confirmed or suspected COVID-19 patient in the past 14 days, as well as whether they have a fever, fatigue, a dry cough, a stuffy nose, a runny nose, throat ache, or diarrhoea. After the information is verified by the authorities, each user is assigned a quick-response code of red, amber, or green. People with a green quick-response code can return to work or school and even travel between different areas and provinces. The local governments also implemented local health codes that are mutually recognised across the whole state.

Second, because timely and accurate tracking of the source of COVID-19 is essential for disease response and control, big data technology is crucial for tracing close contacts. Quick identification and isolation of the source of infectious diseases can enable effective containment of the virus and thus protect the right to the prevention and control of epidemic disease. The Chinese government has used big data technology to identify the life trajectories of infected people and track their history of contact with others, mainly through base station data (operator), payment data (Union Pay and third-party payment institutions), travel data (rail travel, air travel, and accommodation), urban public safety video surveillance systems, and other sources. These data can establish a platform for big data fusion, lock down sources of infection and their close contacts, and provide valuable information for epidemic prevention and control. For example, the official train ticket purchase website 1206 China Railway uses real-name ticket sales and big data in tandem, with local governments and prevention and control agencies providing hundreds of batches of information about those who came into close contact with diagnosed patients on a train. Passenger-related information can be retrieved, including the train number and carriage, and then transmitted to the relevant epidemic prevention department for processing. Telecom operators can track passengers’ trajectories to identify people who appeared in the confirmed patient’s range of travel and accurately pinpoint close contacts. In addition, research showed that up to 84% of patients with COVID-19 are asymptomatic or only have mild symptoms [32]. However, the current systems for controlling transmission rely on patients to report their symptoms to medical professionals, but patients may not be able to recall all of their contacts from the previous few days. As early tracing and quarantining of close contacts is critical for cutting off the transmission chain and limiting the scale of an epidemic [33], developing an effective strategy for precise individual monitoring is becoming increasingly urgent worldwide. Smartphones are widely used devices that are capable of recording users’ spatiotemporal trajectories and social contacts, and Chinese research teams have developed data tracing methods through the WeChat mini-programme Geo WeChat AI System (Geo-WAS) [34]. This mini-programme collects data from users’ voluntary WeChat activities (including time and location labels) and voluntary smartphone-assisted real-world activity history over the previous 14 days (the current maximum incubation period for SARS-CoV-2) to generate an updated quick response code for identification. This technology is highly effective for the early tracing and quarantining of close contacts.

To mitigate the rapid transmission rate of COVID-19, big data technology and other forms of artificial intelligence have been applied to surveillance and digital contact tracing. Several companies, such as Baidu and Alibaba, have developed intelligent epidemic prevention and control systems that consist of an epidemiological analysis system and a transport fever-monitoring system. This system uses artificial intelligence and big data analysis to provide epidemic surveillance, case tracking, information collection, more efficient data mining, comprehensive monitoring, and early warnings about epidemic outbreaks. It provides a reference for policymakers, as it covers decisions regarding infection prevention and control, medical resource allocation, border control, and official travel advice. This technology can also indicate the risk of a virus outbreak and cases coming from other regions [35]. Some companies have developed ‘AI close-contact catchers’ to trace confirmed and suspected cases and investigate close contacts. This system is based on surveillance video network data and uses core algorithms to obtain the trajectory of target personnel within the surveillance range and quickly locate close contacts to achieve rapid positioning, investigation, and control of the spread of the virus [36].

### 4.2. Protecting the Right to Treatment Using Medical Big Data and AI Technology

Patients’ personal information, occupational information, home address, medical treatments, and medical histories are collected and stored in hospitals. Big data, such as clinical symptoms and test results, medical records, treatment plans, and other clinical and diagnostic data, are used for diagnosis, suspected case screening, epidemic isolation, population migration control, and follow-up tracking of recovered COVID-19 patients.

Big data supports the intelligent development of public health fields, such as medical services, new drug development, medical research, and medical management in numerous ways.

First, in the field of medical services, big data technology can be used to treat COVID-19 patients more conveniently and efficiently. Shared data on the diagnosis, treatment, and management of COVID-19 can improve the treatment of other patients [32].

Second, the National Health and Care Commission and Tencent jointly created a National Fever Clinic Map, with WeChat adding a ‘Medical Health Entrance’ applet for online consultations, registration, application for physical examinations, and nucleic acid tests. Since 27 January 2020, the map has facilitated queries to more than 13,000 hospitals, including 11,594 fever clinics and 1512 medical treatment fixed-point hospitals [37].

Third, many hospitals and clinics have launched online or remote consultation to enable patient consultation in a timely and effective manner. The online mental health services being offered during the COVID-19 epidemic are a successful example of this, as they have supported the development of China’s public emergency interventions [38]. However, online consultation cannot solve many practical issues.

### 4.3. Protecting the Right to Health Education and Information Using Big Data Technology

Since 26 January 2020, to help realise citizens’ right to health education and information, some companies have increased national user healthcare services and opened a national COVID-19 outbreak dynamics zone online. This special area includes an ‘expert interpretation’ section, where Tencent’s team of experts has released about 400 original anti-epidemic science and disinformation articles, diagnostic and treatment progress interpretations, popular expert answers, popular science livestreams, and more than 60 popular science videos [37].

To help realise villagers’ right to health education and information, several rural health centres have partnered with various companies to provide free epidemic prevention knowledge in those areas. One health centre in Lingxia City, Gansu province, co-operates with Alibaba Epidemic Prevention and Control Big Data Centre to provide a free epidemic prevention outbound call service operated by robots. This health inquiry service, which can be used by all migrant workers returning to their hometowns within the jurisdiction, asks whether they have a fever, a cough, diarrhoea, vomiting, or other physical discomfort, and provides health education and supervision for epidemic prevention and control. It also reminds the returnees to self-isolate for 14 days, wash their hands frequently, and wear a mask daily for protection [36].

## 5. Impacts of Protecting the Right to Health with Big Data Technology in China: Pros and Cons

### 5.1. Positive Impacts

The positive impacts of protecting the right to health with big data technology during COVID-19 prevention and control in China are discussed below.

**Figure 2:**
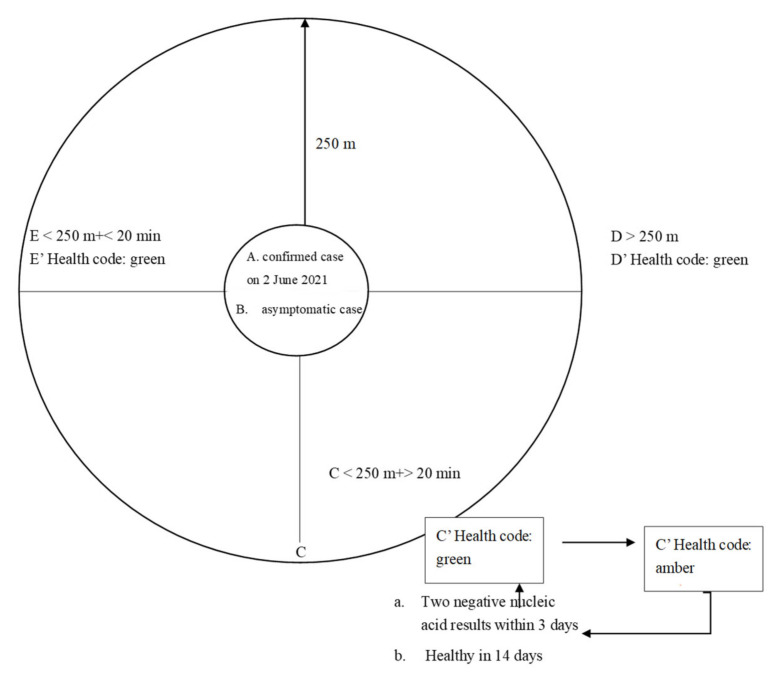
The working mechanism of the Yue Kang code.

First, it enabled precise epidemic prevention and control. Though the officials did not report the detailed rules for the colour change of the Yue Kang code (health code of Guangdong province), certain media reported that the Yue Kang code adjusted the discolouration rule on 2 June 2021 according to the super-contagious property of the Delta variant of the coronavirus. In Figure 2, the Working Mechanism of the Yue Kang Code based on the new discolouration rule is shown as an example to illustrate how it achieves close tracking and supervision of key populations to ensure precise prevention and control using mobile communication big data.

Individuals in group C who were within a 250 m radius of places with confirmed cases (individuals in group A) and asymptomatic cases (individuals in group B) on 2 June 2021 and who stayed there for more than 20 min on the same day are the key populations, and their health code changed from green to amber. Individuals in this group received messages from the police concerning calls to take a nucleic acid test [39]. It was reported that, among the 167 cases in the Guangzhou transmission chain, 53 cases were found to be asymptomatic through the above measures, accounting for 31.7%. It can be said that this is a very important innovation, and it is more targeted, thus improving the efficiency of epidemic prevention and control [40].

Local governments have logs of an individual’s travel history and any possible contact with a confirmed or suspected COVID-19 patient in the past 14 days through the technology of epidemiology, surveillance, and big data. On 8 June 2021, the Guangzhou Municipal Health Commission published a list of 144 places or communities containing infected persons from 21 May to 6 June 2021 [41]. The commission called on those who had recently visited high-risk areas to take the initiative to report their situation and take a nucleic acid test.

Second, big data technology improved the efficiency and accuracy of the diagnosis and treatment of new coronavirus pneumonia. For example, COVID-19 CT image analysis technology is based on deep learning algorithms, uses 5000 CT images for training, and has been tested in many hospitals in China. This technology identifies features of coronavirus pneumonia through CT scans with an accuracy of approximately 96%, and the entire test takes three to four seconds. The detection speed is at least 60 times that of manual detection; this can improve the efficiency of the virus detection program while maintaining a high accuracy rate. Currently, more than 160 public institutions in China are using this technology. The new coronavirus whole-genome sequencing analysis solution based on the artificial intelligence algorithm is used to diagnose the new coronavirus. This technology includes virus gene data scanning, evolution analysis, protein structure analysis, and diagnostic reports. The diagnosis of the new coronavirus can be completed within 14 h, and more than 20 people can be tested at the same time. The average detection time for each sample is only roughly half an hour, which is much lower than the two-hour polymerase chain reaction detection under normal circumstances. This solution can also help disease control centres, hospitals, clinics, and laboratories to improve nucleic acid testing capabilities [42].

### 5.2. Negative Effects

China has used a data-based approach to protect the right to health during the COVID-19 period, and although it has successfully supported the containment of the virus, it also risks infringing on individual privacy rights because of the poor protection of privacy and individual information in China given the great privacy challenges faced during the process of data collection, storage, control, and disclosure [43].

First, during the process of data collection, information is sometimes over-collected during epidemic prevention and control. Governments hope to collect increasing amounts of information for precise and effective epidemic prevention and control. Apps, such as WeChat, have collected personal information, such as each user’s name, address, ID number, mobile number, sex, birth date, nationality, residence address, registered permanent residence, place of birth or origin, health situation, travel history, and close contacts. These are identifiable private personal data, and thus, massive information collection may threaten data privacy [44]. The collection of such irrelevant information is not directly related to circulation and tracking and therefore exceeds the scope of the information that should be collected, violates the principle of proportionality, and increases the risk of privacy infringement. Furthermore, there is some confusion among information collection subjects and controllers. In accordance with Article 38 of the PRC Emergency Response Law, Article 20 of the PRC Regulations on Disclosure of Government Information, Article 25 of the PRC Emergency Regulations on Public Health Emergencies, and Article 30 of the PRC Law on the Prevention and Treatment of Infectious Diseases, local governments at or above the county level, their relevant departments, and professional institutions have the power to collect information about emergencies, and both natural and legal persons are obligated to report what they know. Thus, government departments, professional organisations, neighbourhood committees, village committees, relevant units, and other public authorities have the power to collect emergency information. However, in practice, many other subjects participate in information collection, such as major technology companies in the telecommunications industry, which have been encouraged to do so by the governments because they can use big data technology to analyse and predict diagnosed and suspected persons, the flow of close contacts, and other key groups. In addition, public service units, such as hotels, theatres, restaurants, shopping malls, and other public places, are authorised by the Joint Prevention and Control Agency of the State Council to collect information. Community organisations, various employers, universities, and app developers are also directly involved in information collection activities [43].

Second, during the data control and storage process, citizens’ personal information has not been effectively managed, and personal information has flowed into the hands of third parties in some cases [45]. In one case, a street community worker in Anshun city sent personal information from a community epidemic prevention and control programme to friends. This information spread rapidly via WeChat, resulting in harm. The Anshun Public Security Agency imposed a fine of 5000 yuan and ordered 15 days of administrative detention according to the PRC Public Security Administration Penalty Law [46]. Moreover, there is also a lack of effective safety management in information control and storage. To increase the speed of information exchange and the completion of work, many epidemic prevention and control documents are transmitted through instant communication tools, such as WeChat and QQ, and staff often use these tools to verify flow adjustment information. The use of instant communication tools has increased the speed of information circulation, but there are drawbacks. There was another case that involved the violation of privacy, where the Jian Kang Bao (Health Bao) programme in Beijing made omissions in protecting individual privacy. Using this app, people could find photos of another person and the results of their latest nucleic acid test when entering the name and ID number of that person. Criminals have profited by selling personal photos and other information from the app [47].

Third, improper information disclosure poses a great risk to personal privacy. According to Article 15 of the Regulations of the PRC on Disclosure of Government Information, administrative agencies shall not disclose government information involving company secrets or private information. However, if a third party agrees to the disclosure or if the administrative agency believes that non-disclosure will significantly harm the public, it shall be disclosed. This clause is meant to balance public interest with personal privacy, as administrative agencies are allowed to disclose the neighbourhoods and locations where people have been diagnosed to protect the right to health and to aid epidemic prevention and control. During the early stage of epidemic prevention and control, personal information was not desensitised in the process of data disclosure. For example, it was disclosed that a person named Wang XX, her husband Cai XX, and her daughter Cai XX lived in a particular community, making it easy to identify a specific person. This could have led to discrimination against the parties involved [48].

## 6. Institutional Cause of the Impacts of Big Data Technology Applications for Fighting against COVID-19 in China

### 6.1. Institutional Capacity and Big Data Technology Reinforce Each Other When Fighting COVID-19

The current coronavirus pandemic offers a timely reminder of the linkage between the epidemic and its social determinants and institutional causation, and the notion of “social epidemiology” helps to explain the drastic variations in the health achievements of different societies [49]. The factors responsible for successful pandemic responses have been a state’s capacity, social trust, and leadership. Countries with all three—a competent state apparatus, a government that citizens trust and listen to, and effective leaders—have performed impressively, limiting the damage they have suffered. Countries with dysfunctional states, polarised societies, or poor leadership have done badly [50].

It might be useful to understand the interplay between the institutional capacity and big data technology that inform the actions taken towards the prevention and control of COVID-19 in China by reference to the explanatory paradigm of institutional capacity. According to Potter, institutional capacity refers to the ability of institutions to perform their assigned tasks, which can be examined with reference to factor analysis and expressed through the formula below:

(1) $$IC=\frac{Institutional\;Goal}{\left(u=\frac{1}{1-u}\right)\left(S=S_{n}\right)\left(A=Ar\;var.\;As\right)\left(D=\frac{1}{1-a}\right)}$$

Institutional capacity (*IC*) may be seen as a function of a particular institutional goal that is affected by the following factors: institutional purpose (*U*) concerning the institutional goal, the effects of location (*S*) on the understanding of the institutional goal, the effects of formal and informal orientation (*Ar* and *As*) regarding how the goal is to be pursued, and the extent of institutional cohesion (*D*) in the organisational structure and behaviour [51].

For the discussion of big data technology, the factor of institutional cohesion is of key importance in light of the governance and bureaucratic system of the PRC. Understanding the substantive dynamics of public health initiatives and government measures, such as big data technology applications in China through the paradigm of institutional capacity, may help to examine how relational factors, especially the factor of institutional cohesion, including organisational and functional perspectives, determine the performance of the government in the prevention and control of the COVID-19 pandemic. In the case of China, organisational perspectives—e.g., on the responsibilities of different organisations and their accountability to their superior authorities—and the efficiency of the allocation and use of resources and big data, together with functional perspectives—such as the effectiveness and methods of communication, organisational symmetry, the ability to enforce rules and directives together, and big data technology applications—are the main social determinants of the results of COVID-19 prevention and control. More specifically, the high degree of institutional cohesion enables the government to collect data more accurately and effectively for disease control and prevention, which makes the big data analysis more powerful and effective, and a strong big data technology, in turn, further reinforces the institutional cohesion of the government bureaucracy by facilitating the work of the institution to perform their assigned tasks with the help of data and technology. The data collected by the government can be used for legitimate purposes, such as disease control and crime prevention; however, how to ensure that the personal data be legitimately collected, used, and kept without being abused remains a critical question.

Nevertheless, the efficiency of COVID-19 prevention and control in China has also relied on extensive testing, honest reporting, and the willing cooperation of a well-informed public. In short, the institutional capacity, along with other social determinants, influences the performance of governments in fighting coronavirus and protecting the right to health during the pandemic.

### 6.2. Individualism vs. Collectivism

The history of the right to health is framed as a collective action regarding the health of the population [52], and issues related to disease control were considered chiefly in connection with maintaining and augmenting a healthy population, and thus, in terms of their significance for the political and economic strength of the state [53]. In other words, public health initiatives provided by the government as a collective action were motivated by an expansion in the regulatory power of the state rather than individual concerns about the right to health. Threat to the health of individuals, such as transmissible disease, has been a social concern throughout human history, and thus, the idea of a social interest theory of rights was adopted to recognise that the identification of the interest in which the right to health is grounded is not considered to be essential, basic, natural, or determinate, but to be the product of social process [9].

However, the right to privacy has often been treated as a private right [54,55]. Compared with the right to health, which is considered in terms of public health or interest, the right to privacy is often restricted to maintain public health. Chinese society emphasises economic, social, and cultural rights, as well as collective rights, more so than civil rights and individual rights [56]. Moreover, the lack of legislation to protect personal information and privacy, the weak awareness of personal information and privacy protection, and the insufficiency of protection systems for personal information and privacy are also major reasons for the violation of personal privacy with the use of big data technology.

## 7. Conclusions

Even prior to 2020, China was using big data to mitigate the spread of COVID-19 within the country. Smartphone technology and data gathered from the internet and other sources have helped China to track infected people and their close contacts, which has considerably aided the identification of those who need to be isolated. Although big data technology is not sufficiently advanced to solve all COVID-19-related problems, China has used a data-based approach to fulfil individuals’ health rights. The usefulness of big data in combating the COVID-19 pandemic cannot be ignored. An efficient and precise response is crucial to containing the spread of SARS-CoV-2, and technology is essential in attaining this goal. China has orchestrated some of the most successful efforts to contain the COVID-19 pandemic using tracking applications, epidemic outbreak monitoring, trend forecasting, online consultations, and the allocation of medical and health resources via big data and other technologies. China’s efforts to protect the right to health using big data technology are worth promoting.

However, the role of big data technology in protecting the right to health cannot be overstated. Facing the challenges to privacy raised using big data, policymakers should reduce the negative effect of this technology on privacy. The Chinese government should balance the right to health and privacy requirements; improve its legal systems to protect personal information and privacy, including making liability easier for wronged individuals; and enhance the personal information and privacy protection awareness of administrators and the public. Chinese policymakers are cognizant of the advantages and security of big data technology. The main aims of the Data Security Law of the PRC passed on 10 June 2021 are to regulate the handling of data, ensure data security, and promote the development and exploitation of data; its aim is not to protect the right to privacy.

Based on the literature and the above research, this study proposed concrete remedies for the violation of individuals’ right to privacy. First, the PRC Personal Information Protection Law and other laws that are meant to protect and strengthen data privacy should be prioritised. This will send a signal to government bodies that they must secure individuals’ collected personal data according to official protocols. Second, citizens must be able to take legal action against officials who have revealed their personal information, and courts should ensure that such cases are heard and resolved promptly. Such initiatives will help to strike a proper balance between the right to health and the right to privacy.

If meaningful technoscientific advances can be reached alongside a respect for individual rights, this progress will be stronger and more durable [57]. Human rights are ultimately the core and goal of technoscientific development.
